# Prolactin secreting pituitary neuroendocrine tumors treated by dopamine agonists: predictors of response

**DOI:** 10.3389/fendo.2025.1664621

**Published:** 2025-09-09

**Authors:** Chiara Mele, Marco Zavattaro, Rosa Pitino, Martina Romanisio, Alice Ferrero, Sara Sturnia, Sara Catenazzi, Federica Rosmini, Sabrina Baldi, Paolo Marzullo, Gianluca Aimaretti, Flavia Prodam, Marina Caputo

**Affiliations:** ^1^ Endocrinology, Department of Translational Medicine, Università del Piemonte Orientale, Novara, Italy; ^2^ Department of Radiology, “Maggiore della Carità” Hospital, Novara, Italy; ^3^ Department of Health Sciences, Università del Piemonte Orientale, Novara, Italy

**Keywords:** prolactin, pituitary adenomas, PitNETs, dopamine agonist, resistance

## Abstract

**Purpose:**

To date, no specific criteria have been clearly established to predict the response to dopamine agonists (DA), and a universally accepted definition of DA resistance remains lacking. This study aimed to analyze the clinical, hormonal, and radiological characteristics of patients with prolactin (PRL)-secreting PitNETs, also known as pituitary adenomas, treated with DA, in order to identify potential predictive factors of hormonal and radiological response to medical therapy.

**Methods:**

This retrospective cohort study included 62 patients consecutively admitted to our institution over a 20-year period (2004 – 2024). Seven patients underwent transsphenoidal surgery as first-line treatment before starting DA therapy. Demographic, clinical, hormonal, and radiological data were collected at diagnosis and during follow-up (6, 12, and 24 months, and at the last visit). DA resistance was defined as the failure to normalize PRL levels and to achieve at least a 50% reduction in the tumor’s major diameter or volume.

**Results:**

The median age at diagnosis was 37 years (IQR 26.5 – 45.3), with a male-to-female ratio of 1:1.7. Microprolactinomas were observed in 48.4% of patients. All patients were treated with cabergoline (median dose 1.0 mg/week) and followed for a median of 84 months (IQR 35.3 – 114.0). Macroprolactinomas were more frequent in males, who also showed higher baseline PRL levels. Early PRL response to DA treatment was a significant predictor of long-term hormonal response, independent of sex, age, and DA dosage (OR = 11.29; 95% CI 1.10 – 60.74). Tumor response assessment revealed low agreement between classifications based on diameter versus volume reduction. Diameter-based evaluation was more effective in identifying clinical responders at 6 months and at final follow-up, while volumetric measurements provided greater accuracy at 12 and 24 months.

**Conclusion:**

Normalization of PRL levels is a practical and reliable predictor of treatment response. A combined radiological assessment using both tumor diameter and volume is advisable: diameter offers greater insight in the early stages, while volume becomes more informative in the mid- to long-term follow-up. In patients with persistently elevated PRL levels and lack of radiological response, alternative management strategies—including surgical resection—should be considered, especially in light of recent evidence supporting the cost-effectiveness of surgery in enclosed prolactinomas.

## 1 Introduction

Prolactin (PRL)-secreting adenomas are pituitary neuroendocrine tumors (PitNETs, also known as pituitary adeno-mas) derived from lactotroph cells and represent more than 30% of all pituitary adenomas and up to 60% of function-al pituitary adenomas both in women and men (1, 2).

The prevalence of PRL-secreting adenoma is approximately 50 per 100,000 and the incidence is 3 – 5 new cases/100,000 individuals/year. Microprolactinomas (<10 mm in maximal diameter) are the most frequent subtype. During premenopausal age, microprolactinomas are more frequent among women, with a female to male ratio of 5:1 to 10:1, whereas after menopause the ratio equalizes (3). On the contrary, macroprolactinomas are more frequent and aggressive in males than females (4, 5). Gender differences in tumor behavior could involve several molecular mechanisms, in particular the estrogen-receptor pathway (4, 6), added to a diagnostic delay secondary to subtle or uninvestigated symptoms (i.e., erectile dysfunction and decreased libido).

PRL-PitNETs are treated with surgery or dopamine agonists depending on adenoma size, clinical factors and patient preference. Medical therapy with dopamine agonists (DA), mainly with cabergoline, has been historically considered the first line therapy since it is an effective option, resulting in normalization of prolactin serum levels, adenoma shrinkage and gonadal function restoration (7). In microadenomas, patient preference for active surveillance without DA with or without hormonal replacement therapy could be considered depending on age, menopause, and hypogonadism stage. On the other hand, in patients with macroprolactinomas, therapy with DA is usually suggested as i) these tumors could grow becoming aggressive, particularly in males, in whom invasion of cavernous sinus is frequently observed already at diagnosis; ii) evident symptoms related to increased PRL levels or tumor compression are present.

Surgical resection of microprolactinomas and well-circumscribed macroprolactinomas (Knosp grade 0 and 1) or in patients with intolerance or resistance to DA by an experienced neurosurgeon offers a high chance of cure, is cost-effective and avoids long-term DA treatment, thus entering in the 2024 Consensus Statement (9). Long-term remission is reported in about 83% of microprolactinoma and 60% of macroprolactinoma after surgery (9–12).

Indeed, surgical option could be considered when the medical treatment is unsatisfying, due to intolerance or resistance. DA resistance is defined as the failure to normalize PRL levels and to achieve at least 50% tumor size reduction on the maximally tolerated doses of DA, after at least 6 months of therapy (7). Resistance is more frequent in macroprolactinomas, invasive tumors and male patients (13).

However, the definition of DA resistance is debated since which radiological size should be taken into account (diameter, surface area or volume) and which targets of PRL levels should be to reached during medical therapy have not been pointed out. Moreover, the maximum dose of a DA, or whether gender differences exist, before classifying a patient as resistant has not clearly specified (7).

The aim of this study was to evaluate the clinical, radiological and hormonal characteristics in a cohort of PRL-secreting pituitary adenomas to identify potential clinical predictive factors of hormonal and radiological response during treatment with cabergoline, in the perspective of ameliorating the management and give insights to define precociously who is a candidate to surgery.

## 2 Patients and methods

We searched the electronic medical records of a tertiary care institution (Neuroendocrinology Unit, “Maggiore della Carità” University-Hospital, Novara, Italy) for patients with PRL-secreting pituitary adenomas over the past 20 years (2004 – 2024).

**Figure 1:**
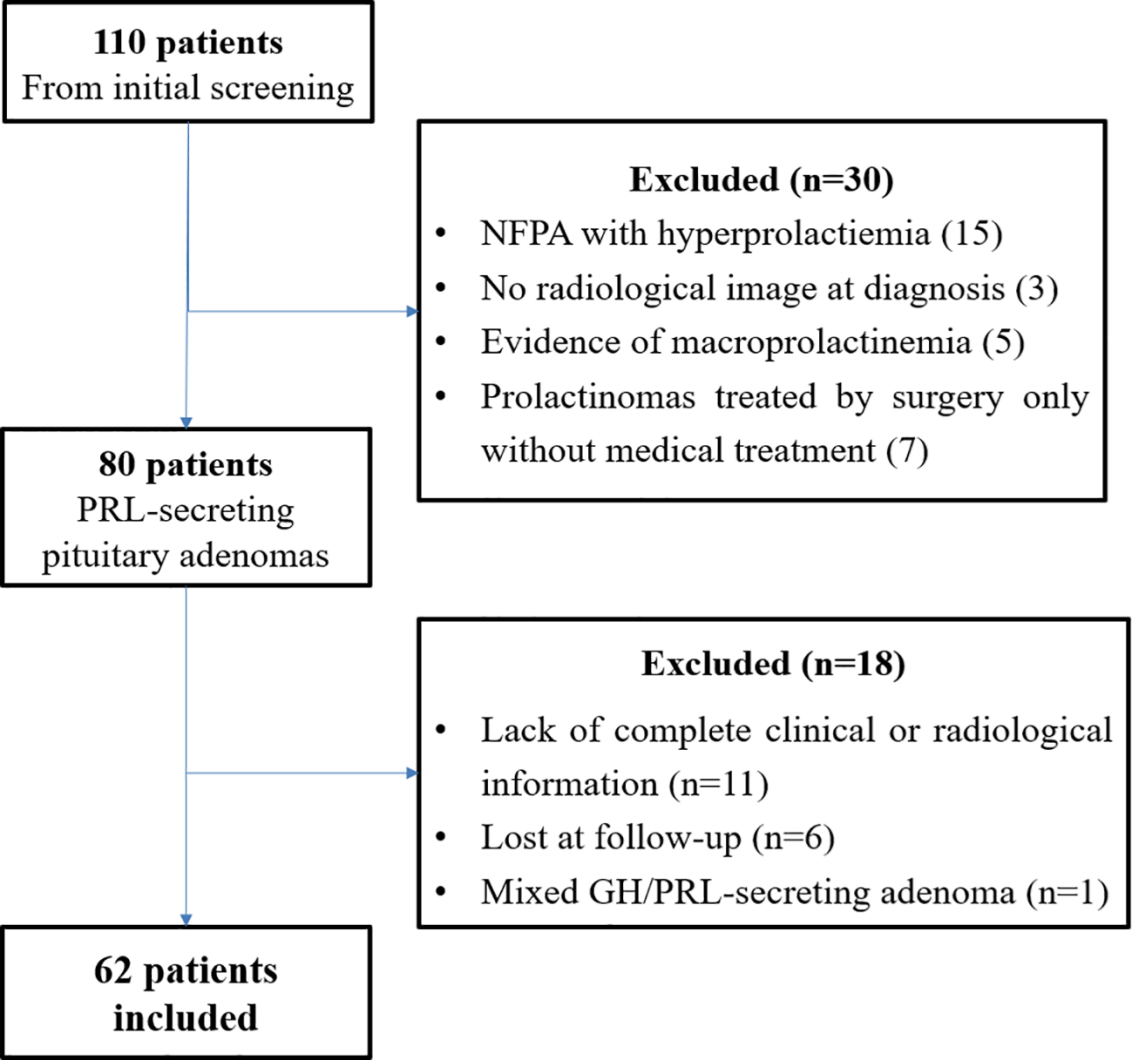
Flowchart of participant selection and inclusion in the study cohort.

From the initial screening of medical records, 110 patients were identified. After preliminary data review, 80 patients were deemed potentially eligible. Following a second, more detailed screening, an additional 18 patients were excluded: 11 due to incomplete clinical or radiological information, 6 were lost to follow-up, and 1 patient had a mixed GH/PRL-secreting adenoma. Consequently, 62 patients were ultimately included in the study (**Figure 1**).

We collected the following data:

- demographic and clinical characteristics: age, gender, symptoms at diagnosis (compressive symptoms as visual deficit; galactorrhea; oligo-amenorrhea or erectile dysfunction, loss of libido, gynecomastia; metabolic (glucose alterations, dyslipidemia), neoplastic, cardiac (valvulopaties), and bone comorbidities at diagnosis and during follow-up;
- hormonal characteristics: PRL level at diagnosis and during follow-up (6 months, 12 months, 24 months and last follow-up); pituitary function at diagnosis and during follow-up; hormonal replacement therapy; time encompassed between diagnosis and restoration of gonadic function after DA treatment;
- treatment: DA medication and maximal dose, treatment starting date, total duration of DA treatment; seven patients underwent transsphenoidal surgery as first-line treatment before starting DA;
- radiological (MRI) characteristics: prolactinoma major diameter (mm) and volume (mm3) at baseline and different time points (6 months, 12 months, 24 months and last follow-up); invasion of nearby anatomical structures (cavernous sinus, optic chiasma).

Exclusion criteria were i) co-secretion of growth hormone; ii) patients without documented PRL values and/or magnetic resonance imaging (MRI) for volumetric analyses.

Serum prolactin (PRL) levels were measured using a chemiluminescent immunoassay (CLIA), which remained the standard method employed in our laboratory throughout the study period. The specific assay platform used was the ADVIA Centaur Prolactin assay (Siemens Healthcare Diagnostics Ltd, Camberley, Surrey, UK), and measurements were performed according to the manufacturer’s instructions. Sex-specific reference ranges in our laboratory were: 2.8 – 29.2 ng/mL for women and 2.1 – 17.7 ng/mL for men. While minor updates in assay calibration or instrumentation may have occurred during the 20-year study period, the analytical methodology remained consistent, and all values were interpreted according to the reference ranges applicable at the time of measurement.

Macroprolactinemia was systematically excluded in all patients. The evaluation was performed using polyethylene glycol (PEG) precipitation, the standard screening method for macroprolactin detection. Only patients with true hyperprolactinemia—defined by the predominance of monomeric prolactin after PEG precipitation—were included in the analysis.

Tumor volume was calculated using the ellipsoid formula: Volume = (π/6) × height × width × depth, with all three dimensions derived from MRI images. Measurements were performed manually on contrast-enhanced T1-weighted MRI sequences. No dedicated volumetric software was employed; all measurements were based on radiological reports and direct review of imaging data by an experienced neuroradiologist.

DA resistance was defined as the failure to achieve almost one parameter among: at least 50% tumor major diameter shrinkage; at least 50% tumor volume shrinkage; failure to normalize PRL levels (<20 ng/mL) after at least 6 months of medical treatment at the maximum tolerated dose. Tumor volume and diameter were reevaluated by an expert neuroradiologist.

The presence of hypopituitarism was defined according to guidelines (14).

All procedures were in accordance with the 1964 Helsinki declaration and its later amendments. The retrospective study was approved by the local ethical committee.

### 2.1 Statistical analysis

Data were expressed as median and interquartile range, absolute number and percentage. Data points not normally distributed, obtained by the Shapiro–Wilk test, were log-transformed to improve the symmetry and homoscedasticity of the distribution.

For comparative analyses between two independent groups, Student’s t test for normally distributed continuous variables or Mann–Whitney U-test for not-normally distributed continuous variable were used. Comparisons between in-dependent dichotomous or categorical data were assessed by the χ2 test. For comparative analyses in case of two paired groups, Student’s paired t test for normally distributed variables or Wilcoxon test for not-normally distributed variables were used. In case of three or more groups, ANOVA for paired data or Friedman test were applied.

Univariate linear regression analysis was used to test association between PRL levels or tumor size at baseline and patients’ clinical characteristics. Univariate logistic regression analysis was used to identify variables associated with treatment response.

Multivariable logistic regression models were built to identified independent predictors of treatment response in term of PRL reduction <20 ng/mL and diameter/volume decrease >50%; Odds ratio (OR), 95% confidence interval (95% CI) and related significant values obtained from regression are reported.

To evaluate the agreement between categorical classifications based on tumor diameter and volume reductions, Co-hen’s kappa coefficient (κ) was calculated. The strength of agreement was interpreted according to Landis and Koch’s criteria. The discriminative ability of diameter and volume reductions in predicting clinical response - defined as a reduction in serum prolactin (PRL) levels - was assessed using receiver operating characteristic (ROC) curve analysis. The area under the ROC curve (AUC) and corresponding 95% confidence intervals (CIs) were computed for each metric at 6, 12, and 24 months, as well as at the last follow-up visit.

P < 0.05 was considered as statistically significant. All analyses were performed with IBM SPSS (version 26.0, IBM SPSS Inc).

## 3 Results

### 3.1 Clinical, radiological and hormonal characteristics at diagnosis

A summary of clinical, hormonal and radiological characteristics of patients at diagnosis is reported in **Table 1**. The median age at diagnosis was 37 years (IQR 26.5 - 45.3), with 23 males (37.1%) and 39 females (62.9%), yielding a F/M ratio of 1.7/1.

**Table 1:** Clinical, hormonal and radiological characteristics of patients at diagnosis, overall and divided by gender.

Variables	Total *(n = 62)*	Males (n = 23)	Females *(n = 39)*	*P-value*
Age *(years)*	37.0 (26.5 – 45.3)	44.0 (32.0 - 50.0)	34.0 (22.0 - 42.0)	0.40
Radiology				
Microadenoma	30 (48.4%)	4 (17.4%)	26 (66.7%)	**< 0.0001**
Macroadenoma	32 (51.6%)	19 (82.6%)	13 (33.3%)	**< 0.0001**
Maximum diameter (mm)	9.5 (5.0 - 19.3)	20.0 (15.0 - 30.0)	7.0 (4.0 - 12.0)	**< 0.0001**
Volume (mm3)	1361 (109 - 3120)	2246 (1195 - 7047)	133 (39 - 1026)	**< 0.05**
Cavernous sinus invasion	11 (17.7%)	7 (30.4%)	4 (10.3%)	**< 0.05**
Surgery	7 (11.3%)	3 (13.0%)	4 (10.3%)	0.74
Symptoms at diagnosis	46 (74.2%)	20 (87%)	26 (66,7%)	0.08
Compressive symptoms	14 (22.6%)	9 (39.1%)	5 (12,8%)	**< 0.05**
Visual defect	13 (21.0%)	7 (30.4%)	6 (15,4%)	0.16
Hormonal deficits	18 (29.0%)	14 (60.9%)	4 (10.3%)	**< 0.0001**
Hormones levels				
PRL (ng/ml)	187.4 (80.1 - 779.6)	683.0 (178.0 - 1000)	145.5 (69.0 - 301.0)	**< 0.05**
TSH (mU/L)	1.6 (1.3 - 2.1)	1.5 (1.0 - 1.7)	1.9 (1.5 - 2.5)	0.14
fT4 (ng/dL)	1.2 (0.9 - 1.2)	1.0 (0.8 - 1.2)	1.2 (1.1 - 1.3)	**< 0.05**
Cortisol (µg/dL)	12.4 (8.6 - 17.6)	12.6 (9.1 - 17.6)	9.4 (6.7 - 18.7)	0.61
IGF-I (ng/mL)	152.6 (123.5 - 179.5)	152.6 (123.5 - 179.5)	146.6 (68.8 - 372.0)	1.00
ACTH (pg/mL)	30.2 (16.1 - 38.6)	34.7 (17.9 - 42.6)	17.1 (11.9 - 33.7)	0.09
FSH (mU/L)	2.9 (1.7 - 6.5)	2.1 (1.5 - 5.0)	5.0 (2.1 - 8.5)	0.13
LH (mU/L)	2.1 (0.5 - 8.0)	1.7 (0.8 - 5.9)	2.7 (0.3 - 9.8)	0.89
Dose of DA (mg/sett)	1.0 (0.5 - 1.4)	1.0 (1.2 - 3.1)	0.5 (0.5 - 1.0)	**0.001**

Data are expressed as median and interquartile range (IQR) or absolute number and percentage.

Comparisons between groups were performed with χ2 test for dichotomous or categorical variables, Student's t test for continuous normally distributed variables and Mann-Whitney test for continuous not normally distributed variables.

Significant differences are shown in bold characters.

At diagnosis, 30 patients (48.4%) had microprolactinomas and 32 (51.6%) macroprolactinomas. The median maximum tumor diameter was 9.5 mm (IQR 5.0 – 19.3; range 2 – 73 mm), with a median tumor volume of 1361.0 mm³ (IQR 109.0 – 3120.0). The mean serum PRL level at baseline was 187.4 ng/mL (IQR 80.1 – 779.6). Due to the high prevalence of macroadenomas, compression of the optic chiasm was observed in 13 patients (21.0%), while 11 patients (17.7%) showed evidence of cavernous sinus invasion on MRI. Hormonal deficiencies were also documented at diagnosis: hypogonadism in 35 patients (56.4%), hypocortisolism in 6 (9.7%), and hypothyroidism in 8 (12.9%).

Seven patients (11.3%) underwent transsphenoidal surgery as first-line treatment due to compressive symptoms. All had macroadenomas; no microprolactinomas were surgically treated in this cohort. These patients were subsequently treated with DA, and post-surgical PRL and radiological data were included in the analysis.

All patients received cabergoline therapy, with a median maximum dose of 1.0 mg/week (IQR 0.5 – 1.4). The median duration of follow-up was 84 months (IQR 35.3 – 114.0).

Regarding metabolic profile, most patients had normal glucose metabolism, with a median fasting plasma glucose of 87 mg/dL (IQR 76.7 – 92.7) and HbA1c of 5.5% (IQR 5.3 – 5.7). During follow-up, 2 new cases of type 2 diabetes and 3 cases of prediabetes were diagnosed. Lipid parameters at baseline included median total cholesterol of 202.0 mg/dL (IQR 117.8 – 238.0), HDL 59.0 mg/dL (IQR 45.0 – 69.5), triglycerides 113.5 mg/dL (IQR 78.5 – 172.6), and LDL 120.5 mg/dL (IQR 85.5 – 164.0). One patient was already on statins at diagnosis; during follow-up, 13 patients began lipid-lowering therapy due to worsening lipid profiles.

With respect to bone metabolism, hypovitaminosis D was common, with a median vitamin D level of 16.3 ng/mL (IQR 10.9 – 26.2). Serum calcium was within normal limits (median 9.1 mg/dL, IQR 8.9 – 9.6). Vertebral osteoporosis without complications was diagnosed in 3 patients (11.1%).

During the follow-up, a single case of neoplasia was documented (utero-ovarian carcinoma in postmenopausal woman). Additionally, 10 patients (16.1%) were diagnosed with non-clinically significant atrioventricular valvular disease.

### 3.2 Gender differences

A summary of clinical, hormonal, and radiological characteristics according to gender is presented in **Table 1**. Macroprolactinomas were significantly more frequent in males compared to females (p < 0.0001), who consequently exhibited larger maximum tumor diameters (p < 0.0001) and volumes (p < 0.05) at diagnosis. Consequently, males also demonstrated a higher prevalence of compressive symptoms (p < 0.05), cavernous sinus invasion (p < 0.05), and pituitary deficits (p < 0.0001). Likewise, PRL levels were higher in males compared to females (p < 0.05). Among other pituitary hormones, free thyroxine (fT4) levels were lower in males (p < 0.05), while no significant sex differences were found for other pituitary axes. Regarding treatment response, males showed a significantly higher PRL reduction at short-term follow-up (6 months) compared to females (p < 0.05), and this difference was maintained at 12 and 24 months.

### 3.3 PRL levels

Serum PRL levels at various time points are summarized in **Table 2**. Patients with macroprolactinomas had significantly higher baseline PRL levels than those with microprolactinomas (median 722.7 ng/mL, IQR 391.8 – 1000 vs 96.3 ng/mL, IQR 68.5 – 145.6, p < 0.0001), and accordingly required higher starting doses of DA (median 1.0 mg/week, IQR 1.0 – 3.0 vs 0.5 mg/week, IQR 0.5 – 1.0, p < 0.0001). Univariate linear regression showed that higher baseline PRL levels were positively predicted by male sex (β=0.45, p=0.001), optic chiasm compression (β=0.67, p<0.0001), sphenoid sinus invasion (β=0.43, p=0.002), and tumor size (β=0.80, p<0.0001).

**Table 2:** Hormonal and radiological changes over time.

Variables	T_0_ *(n = 62)*	T_6_ *(n =40)*	T_12_ *(n = 46)*	T_24_ *(n = 42)*	T_LAST_ *(n = 62)*	P-value (paired data)
PRL *(ng/mL)*	187.4 (85.0 - 742.6)	10.5 (4.2 - 22.8)*	7.3 (3.2 - 24.7)*	5.8 (1.3 - 20.2)*	10.8 (2.8 - 19.8)*	**<0.0001**
Maximum diameter (*mm)*	9.5 (5.0 - 18.8)	10.5 (4.3 - 15.0)*	7.0 (4.0 - 12.0)*	5.0 (3.3 - 11.0)*^1^	4.5 (0.0 - 10.8)*^1^	**<0.0001**
Volume *(mm^3^)*	1361 (133 - 2913)	842 (541 - 1217)*	157 (46 - 735)*^1^	137 (28 - 503)*^1^	84.0 (20 - 707)*	**<0.0001**
Δ diameter *(%)*	–	25.0 (0.0 - 44.3)	22.5 (0.0 - 40.0)	37.2 (0.0 - 58.9)*^1^	54.2 (20.4 - 100.0)^1^	**<0.0001**
Δ volume *(%)*	–	70.0 (61.3 - 82.1)	68.2 (38.9 - 85.8)^1^	78.7 (62.8 - 90.8)*^1^	82.1 (63.1 – 96.6)*^1^	**0.002**

*difference vs T0; ^1^difference vs last control.

The comparison between two time points was assessed by paired T-test for normally distributed variables and Wilcoxon test for not normally distributed variables. The comparison among all the time points was performed by paired data ANOVA for normally distributed variables or Friedmann test for not normally distributed variables.Significant differences are shown in bold characters.

Treatment with DA led to a significant reduction in PRL levels over time (p<0.0001).

Among patients with hypogonadism at diagnosis (N = 35), 26 (74.2%) showed recovery of pituitary-gonadal axis function by the last follow-up. Approximately 70% of patients achieved the goal of a PRL decrease <20 ng/mL at 6 and 12 months, with this proportion increasing to 74.3% at 24 months and 74.2% at the last follow-up. Univariate logistic regression did not identify any significant association between successful PRL response, patients’ clinical/radiological characteristics and dose of DA. Indeed, a multivariable logistic regression model was built to identify the independent predictors for successful response of PRL values <20 ng/mL (**Table 3**) throughout the follow-up times. Basal PRL emerged as the only predictor of normalization of PRL levels within 6 months and at the last follow-up visit (OR = 0.99, CI 95% 0.99 - 1.00, p=0.04 for both). In particular, higher PRL levels at baseline were associated with a lower prevalence of PRL normalization. Sex, age, baseline PRL, dose of DA, been micro- or macroadenoma did not represent predictors for PRL response after 12 and 24 months. Notably, early PRL response to DA treatment was a significant predictor of the long term PRL response independently from sex, age and DA dose (OR = 11.29, CI 95% 1.10 - 60.74, p=0.005).

**Table 3:** Multivariable logistic regression model to test the predictors for significant response in term of reduction in PRL levels.

Regression model	PRL <20 ng/mL after 6 months		PRL <20 ng/mL after 12 months		PRL <20 ng/mL after 24 months		PRL <20 ng/mL At the last visit	
	OR (95% CI)	P value	OR (95% CI)	P value	OR (95% CI)	P value	OR (95% CI)	P value
Age	1.03 (0.98 - 1.09)	0.26	0.99 (0.89 - 1.10)	0.81	1.09 (0.89 - 1.35)	0.40	1.01 (0.94 - 1.09)	0.77
Sex	4.72 (0.61 - 36.52)	0.14	1.67 (0.04 - 71.42)	0.79	1.58 (0.03 - 68.53)	0.58	0.31 (0.03 - 3.80)	0.36
PRL T0	**0.99** **(0.99 - 1.00)**	**0.04**	1.00 (0.99 - 1.00)	0.76	1.00 (0.99 - 1.01)	0.91	**0.99** **(0.99 - 1.00)**	**0.04**
Micro/macro	0.70 (0.08 - 5.86)	0.74	2.12 (0.06 - 78.65)	0.68	0.40 (0.02 - 7.09)	0.53	3.08 (0.78 - 12.18)	0.60
Dose of DA (mg/sett)	0.92 (0.50 - 1.69)	0.79	0.004 (0.01 - 2.87)	0.10	0.93 (0.75 - 1.16)	0.54	0.61 (0.23 - 1.62)	0.32

Significant differences are shown in bold characters.

### 3.4 Tumor diameter

Tumor diameter measurements at different time points are reported in **Table 2**. Complete adenoma shrinkage, defined as no residual lesion visible on the last follow-up MRI, was observed in 21 patients, including five cases of macroadenomas. Importantly, none of these patients had undergone prior neurosurgical intervention. Larger tumor diameter at diagnosis was associated with compressive symptoms (OR = 1.07, 95% CI 1.01 – 1.12, p=0.01), optic chiasm compression (OR = 1.17, 95% CI 1.07 – 1.28, p<0.0001), sphenoid sinus invasion (OR = 1.13, 95% CI 1.05 – 1.22, p=0.002), higher PRL levels (β=0.80, p<0.0001), and male sex (β=0.48, p<0.0001).

DA therapy resulted in a significant decrease in tumor diameter over time (p<0.0001) (**Table 2**). A reduction of tumor diameter >50% was achieved by 20.8%, 19.6%, 33.3%, and 54.8% of patients at 6, 12, 24 months, and last follow-up, respectively. Univariate logistic regression did not identify any significant association between tumor diameter reduction, patients’ clinical/radiological characteristics and dose of DA. Multivariable logistic regression model was built to identify the independent predictors for significant response in terms of diameter reduction >50% throughout the follow-up times. Sex, age, dose of DA and tumor diameter at baseline did not represent predictors for diameter decrease during follow-up. Moreover, an early response to DA treatment in terms of diameters decrease >50% did not represents a predictor of long-term diameter response.

### 3.5 Tumor volume

Consistent with tumor diameter findings, DA therapy led to significant tumor volume reduction over time (p<0.0001) (**Table 2**). Higher baseline tumor volume was significantly associated with optic chiasm compression (OR = 1.002, 95% CI 1.001 – 1.003, p=0.04), sphenoid sinus invasion (OR = 1.00, 95% CI 1.00 – 1.001, p=0.04), elevated baseline PRL levels (β=0.50, p=0.008), and male sex (β=0.52, p<0.0001). Tumor volume reduction >50% was observed in 50.0%, 65.4%, 81.8%, and 77.8% of patients at 6, 12, 24 months, and last follow-up, respectively.

Univariate and multivariable analyses found no significant associations between tumor volume response (volume reduction >50%) and clinical/radiological features or DA dose. Similarly to diameter, early volume response did not predict long-term tumor volume reduction.

### 3.6 Agreement between diameter and volume response

Agreement between tumor diameter and volume response classifications was low, as indicated by Cohen’s kappa coefficient (**Table 4**), consistent with observed response rates. ROC curve analyses revealed that diameter-based assessment was more accurate in identifying clinical responders at 6 months and at the final evaluation, whereas volumetric measurements showed greater accuracy at 12 and 24 months (**Table 5**). Clinical response was defined based on serum PRL reduction at all follow-up time points.

**Table 4:** Cohen’s kappa coefficient to test the agreement between diameter and volume response.

Volume and diameter response	Cohen’s k coefficient	P
6 months	0.31	0.08
12 months	0.18	0.11
24 months	0.04	0.75
Last visit	0.23	0.09

**Table 5:** Area under the ROC curve (AUC) and 95% confidence intervals for volume and diameter in predicting clinical response (defined as serum PRL reduction) at 6, 12, and 24 months, and at the last follow-up visit.

PRL response	Diameter AUC (CI 95%)	Volume AUC (CI 95%)
6 months	0.44 (0.15 - 0.73)	0.18 (0.00 - 0.44)
12 months	0.51 (0.27 - 0.75)	0.53 (0.23 - 0.82)
24 months	0.71 (0.43 - 0.99)	0.93 (0.81 - 1.00)
Last visit	0.49 (0.31 - 0.67)	0.27 (0.00 - 0.54)

## 4 Discussion

In recent years, potential predictors of DA efficacy in PRL-PitNETs (also known as pituitary adenomas) management have been investigated (15, 16) without reaching definitive indications.

This uncertainty could result from several reasons, such as the fact that the radiological dimension (diameter, surface area or volume) and target successful values of PRL to achieve during DA therapy are not clearly specified.

In the light of these shadows, this retrospective study aimed to analyze the clinical, hormonal and morphological characteristics of patients with prolactinoma in order to identify clinical predictive factors of successful response to DA, easy to check into the clinical practice.

As expected, the first clinically relevant result is the gender difference in terms of tumor size. In particular, macroprolactinoma was most frequent in males, who had a greater maximum diameter and tumor volume than females at diagnosis. It is known that the diagnosis is generally earlier in females, due to the early onset of amenorrhea (17), however the age was quite similar between gender in our cohort. Further, a specific pathogenesis of prolactinomas in males has been postulated, since they had increased cell proliferation (as measured by Ki-67), cellular atypia, angiogenic and proliferative characteristics, and greater invasiveness (4). In World Health Organization 2017 Classification of Pituitary Tumors, lactotroph adenomas in males were classified as “high-risk” pituitary adenomas (18), as they are less responsive to medical treatment (19). In a cohort of 122 patients with macroprolactinomas, Delgrange et al. demonstrated that tumors in males were more frequently invasive than in females and, even when considering non-invasive tumors only, the median dose of cabergoline necessary to obtain PRL normalization was still significantly higher in them (19). Nevertheless, neither tumor invasiveness nor gender predicted tumor shrinkage, which was more likely to occur in cases of PRL normalization. Male patients have also an increased risk of non-surgical (spontaneous or DA-induced) cerebrospinal fluid rhinorrhea in the presence of an invasive, DA-resistant macroprolactinoma (20). Finally, when cabergoline is withdrawn, the recurrence of hyperprolactinemia is higher in males than in females (21). In line with these previous evidences, in our cohort male sex was associated with both a higher tumor diameter and volume at baseline. Moreover, as demonstrated by other studies (22), males had an earlier clinical and radiological response than female, and these clinical findings were maintained at the long-term follow-up.

Considering the response to the treatment, we evaluated the predictors of PRL levels normalization and tumor shrinkage, in terms of >50% diameter or volume decrease.

According to previous evidences (23), higher PRL levels at baseline were associated with a lower prevalence of PRL normalization. In a prospective study of 71 males with macroadenomas, the likelihood of achieving normoprolactinemia was higher in those with lower prolactin levels and smaller adenomas at presentation. Additionally, lower prolactin levels and tumor shrinkage after 6 months of treatment were predictive of subsequent normoprolactinemia and further tumor shrinkage, respectively (23). Colao et al. evaluated 204 patients with adenomas treated with cabergoline and demonstrated that high basal PRL levels at diagnosis were negative predictors of PRL normalization at 6 months (24). In this context, other studies highlighted that the nadir prolactin level during treatment was the most important predictor of tumor shrinkage (25, 26).

In our study, an early biochemical response to DA treatment represents a significant predictor of long term PRL response. The same results were obtained by Akinduro et al., who demonstrated that reduction in PRL levels was more pronounced in the first 6 months of treatment, with a rate of 86 ng/mL/month in this period, followed by an overall rate of approximately 7 ng/mL per month for the next 6 months, and then 1 ng/mL/month thereafter (22). These data suggest that prolactinomas with failure to achieve normalization of PRL levels, together with no size regression, by 12 months may be considered for alternative management strategies, as recommended by most recent guidelines and consensus statements (8, 9). The biological basis of DA resistance remains poorly understood. Several possible explanations have been suggested, including low affinity of the dopamine receptor to its ligands, low density of the D2 receptors on the lactotroph cell surface, reduced expression of genes involved in D2 receptor signaling (i.e., NGF receptor), impaired balance between the short and long receptor isoforms, and reduced expression of inhibitory G protein that couple D2R to adenylyl cyclase (27, 28). Moreover, a recent study demonstrated that NEK2, whose overexpression significantly promotes pituitary tumor growth and cell proliferation, is upregulated in resistant prolactinomas (29), thereby impairing cellular sensitivity to cabergoline.

Our results did not show significant predictors of tumor shrinkage, in contrast with a previous study which identified male sex and cavernous sinus invasion as potential predictors of partial or complete resistance to treatment (19). This conflicting finding could result from several factors, including the relatively small cohort size, the proportion of male patients, and differences in the definition of tumor shrinkage. In our study, shrinkage was defined as a reduction of ≥50% in either the maximum tumor diameter or tumor volume, which may not fully align with the criteria applied in other studies.

In fact, our concordance analysis revealed a low level of agreement between volume-based and diameter-based classifications, suggesting that the two measures may not be interchangeable in assessing clinical response. Our findings show that the discriminative ability of tumor size metrics in predicting clinical response, defined as PRL reduction, varies over time. Specifically, diameter reduction demonstrated higher accuracy at 6 months and at the last follow-up, whereas volume reduction showed better performance at 12 and 24 months, as reflected by the respective AUC values. These results suggest that diameter may provide an earlier indication of treatment response, likely due to its simplicity and sensitivity to rapid structural changes. However, volumetric assessment appears to better capture longer-term tumor shrinkage, potentially offering a more robust evaluation of sustained therapeutic effects.

While volumetric analysis may provide a more comprehensive estimate of tumor burden, its role in early response assessment appears limited. This may be due to assumptions of regular tumor geometry in volume formulas, reduced sensitivity to minor dimensional changes, and, in some cases, transient cystic changes during DA treatment—particularly in macroadenomas—that can artifactually increase volume estimates. Although such cases were not observed in our cohort, these factors could contribute to the lower accuracy of volumetric assessment at early time points.

Taken together, our data support a complementary use of both metrics in clinical follow-up, with diameter being informative in the early phase and volume becoming more relevant in the mid- to long-term evaluation.

Recent studies focusing on macroprolactinomas (30, 31) have shown that early tumor shrinkage—assessed within 3 to 12 months—is a stronger predictor of long-term response than baseline tumor size or prolactin levels. In our cohort, we did not observe a similar association between early radiological response and long-term outcomes. This discrepancy may reflect differences in patient population, particularly the smaller average tumor size (median diameter: 9.5 mm). These findings suggest that early tumor shrinkage may be a useful prognostic tool primarily in larger macroprolactinomas, and highlight the need for size-adjusted predictive models.

Our study has some limitations that should be pointed out, as follows: first, the small sample size; second, the retrospective nature, which may be associated with selection bias and incomplete data, potentially affecting the generalizability and strength of the findings; third, the inclusion of a subset of patients who underwent transsphenoidal surgery prior to dopamine agonist initiation, which may have introduced confounding factors due to postsurgical modifications in prolactin levels, tumor volume, and treatment responsiveness. On the other hand, a strength of this study is the evaluation of both hormonal and tumoral response, thus overcoming the uncertainties related to the lack of a clear definition of DA resistance. While no predictors of tumor shrinkage > 50% has been found, a predictor of PRL normalization has been identified. Hormonal restoration is an easily assessable endpoint with clinical relevance, and the lack of PRL normalization remains the cornerstone in defining resistance to treatment (32).

In conclusion, our results demonstrate that the only significant predictor of hormonal response in DA-treated prolactinomas was baseline PRL level. Additionally, an early reduction in PRL level was associated with a favorable long-term hormonal response. Regarding radiological response, a complementary assessment using both tumor diameter and volume appears advisable. While diameter provides more informative data during the early phase of treatment, tumor volume becomes increasingly relevant for mid- to long-term follow-up evaluations.

In patients who exhibit persistently elevated PRL levels despite DA therapy at short-term follow-up, it may be reason-able to consider alternative management strategies, including surgical resection—particularly in light of recent evidence supporting the cost-effectiveness and clinical benefits of surgery for microprolactinomas and enclosed macroprolactinomas.

## Data Availability

The original contributions presented in the study are included in the article/supplementary material. Further inquiries can be directed to the corresponding author.
